# Phenotype and Functional Features of Human Telomerase Reverse Transcriptase Immortalized Human Airway Smooth Muscle Cells from Asthmatic and Non-Asthmatic Donors

**DOI:** 10.1038/s41598-017-18429-0

**Published:** 2018-01-16

**Authors:** J. K. Burgess, A. Ketheson, A. Faiz, K. A. Limbert Rempel, B. G. Oliver, J. P. T. Ward, A. J. Halayko

**Affiliations:** 1 University of Groningen, University Medical Center Groningen, Department of Pathology and Medical Biology, GRIAC (Groningen Research Institute for Asthma and COPD), Groningen, The Netherlands; 2 University of Groningen, University Medical Center Groningen, KOLFF Institute, Groningen, The Netherlands; 3 Woolcock Institute of Medical Research, The University of Sydney, Glebe, NSW Australia; 4 Discipline of Pharmacology, Faculty of Medicine, The University of Sydney, Sydney, NSW Australia; 5 University of Groningen, University Medical Center Groningen, Department of Pulmonology, GRIAC (Groningen Research Institute for Asthma and COPD), Groningen, The Netherlands; 6 University of Manitoba and Children’s Hospital Research Institute of Manitoba, Winnipeg, Canada; 7 School of Medical and Molecular Biosciences, University of Technology Sydney, Sydney, NSW Australia; 8 Kings College London, London, UK

## Abstract

Asthma is an obstructive respiratory disease characterised by chronic inflammation with airway hyperresponsiveness. In asthmatic airways, there is an increase in airway smooth muscle (ASM) cell bulk, which differs from non-asthmatic ASM in characteristics. This study aimed to assess the usefulness of hTERT immortalisation of human ASM cells as a research tool. Specifically we compared proliferative capacity, inflammatory mediator release and extracellular matrix (ECM) production in hTERT immortalised and parent primary ASM cells from asthmatic and non-asthmatic donors. Our studies revealed no significant differences in proliferation, IL-6 and eotaxin-1 production, or CTGF synthesis between donor-matched parent and hTERT immortalised ASM cell lines. However, deposition of ECM proteins fibronectin and fibulin-1 was significantly lower in immortalised ASM cells compared to corresponding primary cells. Notably, previously reported differences in proliferation and inflammatory mediator release between asthmatic and non-asthmatic ASM cells were retained, but excessive ECM protein deposition in asthmatic ASM cells was lost in hTERT ASM cells. This study shows that hTERT immortalised ASM cells mirror primary ASM cells in proliferation and inflammatory profile characteristics. Moreover, we demonstrate both strengths and weaknesses of this immortalised cell model as a representation of primary ASM cells for future asthma pathophysiological research.

## Introduction

Asthma is an obstructive respiratory disease that is characterised by airway hyper-responsiveness, airway obstruction and chronic inflammation^1^. Inflammation and structural changes, referred to as ‘airway remodelling’ are important features of asthma^2,3^. One prominent feature in the airway wall of asthmatics is an increased bulk of airway smooth muscle (ASM)^4^.

The ASM bundles are found in the submucosal layer where they wrap circumferentially around the airway^5^. In ASM derived from asthmatic donors compared to ASM cells from non-asthmatic donors *in vitro* cellular hyperplasia, in response to a proliferative stimulus, has been reported^6,7^. The ASM is not alone but connected to a network of extracellular matrix (ECM) proteins. Typically, these proteins act as support structures for the cells, however, they are also biologically active and can influence aspects of the ASM’s cellular functions^8–13^.

The ASM derived from asthmatics produces an altered inflammatory profile of cytokines and chemokines and has altered ECM protein production^13–17^. These changes in the inflammatory profile are thought to potentiate the inflammatory nature of asthma, while the altered matrix is able to feedback back to the cells within the microenvironment to influence other cellular functions. In this study we focussed on investigating certain key cytokines (including Interleukin (IL)-6^18–22^ and Eotaxin-1^23^), growth factors (Connective tissue growth factor (CTGF)^24,25^) and ECM proteins (fibronectin^26^ and fibulin-1^17^) previously reported to be increased in asthma. Interleukin (IL)-6 is a key inflammatory cytokine that has been characterised in asthma. It is mainly produced by innate immune cells, such as macrophages, but it can also be derived from ASM cells, with greater production by cells from asthmatic than nonasthmatic donors^18–22^. Eotaxin-1, an important chemokine in asthma that attracts eosinophils to sites of inflammation in the airways^27^, is also a key difference seen between primary asthmatic and non-asthmatic ASM cells. Chan *et al*. reported asthmatic ASM cells released greater amounts of eotaxin-1 in response to a variety of inflammatory stimuli^23^. CTGF has been shown to be increased in asthmatic patients. With angiogenic and fibrotic properties, the high levels of CTGF can result in airway remodelling of the asthmatic airway^25^. In terms of the ASM, pre-treatment of ASM cells with transforming growth factor-beta (TGF-β) stimulates the release of CTGF. Additionally, asthmatic ASM cells release significantly greater amounts of CTGF compared to non-asthmatic cells^24,25^. Fibronectin is a pro-proliferative ECM protein that is deposited in greater amounts by asthmatic ASM cells, particularly following TGF-β induction^26^. Fibulin-1 is another pro-fibrotic ECM protein that has been shown to be increased in asthmatic serum and bronchoalveolar lavage (BAL) fluid^17^. The same study reported that TGF-β stimulated asthmatic ASM cells deposited increased amounts of fibulin-1 in the matrix than non-asthmatic cells.

These changes in fundamental functional properties of ASM from asthmatics support an important role in disease pathophysiology. The gold standard model for *in vitro* research of ASM in asthma is primary culture of cells from human donors. Primary cells are isolated from ASM bundles – dissected from human lung tissue obtained by lung resection, transplant or biopsy - and expanded in culture^28^. They provide a model for studying *in vivo* cellular responses^29^, however, major limitations in routinely using primary ASM cell cultures include obtaining primary tissue, particularly from asthmatics, and the limited number of cell doublings that ASM cells can undergo before they lose fundamental phenotypic features of ASM, change morphology or undergo senescence.

Immortalisation of primary ASM cells derived from individuals with and without asthma was originally described by Gosens *et al*.^30^. Through stable ectopic expression of human telomerase reverse transcriptase (hTERT), the lifespan of these cells was extended. Moreover, fundamental phenotypic features of the cells were maintained through multiple passages and cell doublings that far exceed that of primary ASM cell cultures, thus providing a versatile resource for experimentation.

Despite their continued use, there remain gaps in understanding of the strengths and limitation of using hTERT immortalised ASM cells for fundamental experimentation. To date no studies have directly compared the features of hTERT ASM cells to those of donor-matched primary ASM cell lines. In the current study we directly address whether hTERT immortalised ASM cell lines retain unique features of parent primary ASM cells, using cells derived from asthmatic and non-asthmatic donors.

## Materials and Methods

### Primary Airway Smooth Muscle Cell Isolation and culture

Approval for experiments with human lung tissue was provided by the Ethics Review Committee of the South West Sydney Area Health Service, St Vincent’s Hospital Sydney, Strathfield Private Hospital, Royal Prince Alfred Hospital and the University of Sydney Human Research Ethics Committee, as well as the South East London Research Ethics Committee (REC reference number: 10/H0804/66), and the Human Research Ethics Committee of the University of Manitoba. All clinical procedures conformed to the standards set by the latest Declaration of Helsinki.

Primary human ASM cells were obtained through dissection of donated lung tissue following deep endobronchial biopsies from volunteers who provided written informed consent, as described previously^6,17^. Healthy volunteers were selected based on a life-long absence of respiratory symptoms, and, where possible, lung functions within normal limits. Moderate asthmatics were selected based on the presence of typical symptoms over at least two years, a minimum of 12% reversibility, FEV1 < 80% of predicted for age, and/or a bronchoconstrictor response to a methacholine challenge of <8 mg/ml. Patients currently receiving leukotriene receptor antagonists, smokers and pregnant/lactating females were excluded. One non-asthmatic (healthy) ASM cell line was sourced from a lung donated for transplantation. Though it was deemed unsuitable for transplant, it was not from an individual with any known lung pathology (Table 1).

**Table 1:** Patient Characteristics.

Patient^#^	Age	Sex	Health Status	Sample	Country of Origin	Primary or Immortalised
1	22	F	Healthy	Biopsy	Australia	Both
2	69	M	Healthy	Biopsy	Australia	Both
3	21	M	Asthmatic	Biopsy	Australia	Both
4	48	M	Healthy	Donor lung	Australia	Both
5	45	M	Asthmatic	Biopsy	Australia	Both
6	33	M	Asthmatic	Biopsy	Australia	Both
7	36	M	Asthmatic	Biopsy	United Kingdom	Only Immortalised
8	39	M	Asthmatic	Biopsy	United Kingdom	Only Immortalised
9	37	M	Asthmatic	Biopsy	United Kingdom	Only Immortalised
10	37	F	Healthy	Biopsy	United Kingdom	Only Immortalised
11	28	F	Healthy	Biopsy	United Kingdom	Only Immortalised

Cells were established at both the University of Sydney and Kings College London. Primary ASM cells underwent hTERT immortalisation at the University of Manitoba as previously described^30^. The phenotype of all ASM cell lines established in both laboratories was confirmed using immunofluorescence and light microscopy to identify the distinct hill and valley pattern of growth. Cells were stained with antibodies against α-smooth muscle actin and calponin while omission of the primary antibody was used as a control, as previously published^31,32^.

Primary ASM cell cultures were only used between passage numbers 2 and 8, while corresponding hTERT ASM cell lines were used up to passage number 20.

For all cultures, ASM cells were seeded into multi-well plates at a density of 3.2 × 10^3^ cells/ml in 10% foetal bovine serum (FBS) (DSKH, Melbourne, Australia), 1% antibiotics supplemented Dulbecco’s modified Eagle medium (DMEM) (Life Technologies, Carlsbad, CA, USA) for 24 hours. Aspiration of seeding media was followed by the addition of quiescing media (0.1% bovine serum albumin (BSA) (Sigma Aldrich, St Louis, Missouri, USA), 1% antibiotics in DMEM), which was left for three days. Stimulations appropriate for the outcome measure were then added.

### Proliferation

The proliferation of the ASM cells in the presence of 5% FBS was assessed via manual cell counting on days 0, 3, 5 and 7 post treatment, as previously^6^.

### Detection of IL-6 and Eotaxin-1 by ELISA

ASM cells were seeded in 6 well plates at 3.2 × 10^3^ cells/ml in 10% foetal bovine serum (FBS) (DSKH, Melbourne, Australia), 1% antibiotics supplemented Dulbecco’s modified Eagle medium (DMEM) (Life Technologies, Carlsbad, CA, USA) for 24 hours, followed by quiescing three days in 0.1% bovine serum albumin (BSA) (Sigma Aldrich, St Louis, Missouri, USA), 1% antibiotics in DMEM prior to being treated with 10 ng/ml IL-1β or 10 ng/ml TNF-α. Supernatant samples were collected over a 24 hour time course (0, 8, 16 and 24 hours) and stored in aliquots at −20 °C until analysis. IL-6 (BD Biosciences, Franklin Lakes, NJ, USA) and eotaxin-1 (R&D Systems, Minneapolis, MN, USA) were detected with an enzyme linked immunosorbent assay (ELISA) kit, according to the manufacturers’ instructions.

### Detection of cell associated CTGF by ELISA

ASM cells were seeded in 96 well plates as described above, then treated with 10 ng/ml TGF-β1 for 24 hours. Supernatant was removed, the plate was allowed to dry overnight, then stored at −20 °C in an airtight container until CTGF was detected using a cell surface ELISA technique as described previously^33^.

### Detection of Fibronectin and Fibulin-1 by ELISA

ASM cells were seeded into 96 well plates as described above then treated with 10 ng/ml TGF-β1 for 48 and 72 hours. Supernatant was removed and wells were washed with PBS/0.05% Tween 20 three times. Thereafter, the ECM was exposed by treating the cell layer with ammonium hydroxide (Sigma Aldrich) for 30 minutes at 37 °C. After viewing the plate under a light microscope to confirm cell detachment, the plate was stored in PBS at −20 °C until required.

Fibronectin (FN) and Fibulin-1 (FBLN-1) ELISAs were conducted as described previously^34–36^. Briefly, the FN ELISA included standards (BD Biosciences) serially diluted in 0.1% BSA 1% antibiotic supplemented DMEM (2000 ng/ml to 31.25 ng/ml) added in duplicate to the plate which was left at 4 °C overnight on an orbital mixer. The following day both the FN and FBLN-1 ELISA plates were washed, blocked and FN primary antibody, mouse anti-human plasma fibronectin C-Terminal (Chemicon, Billerica MA USA) diluted 1:500 or FBLN-1 primary antibody (Santa Cruz) diluted 1:3000 or isotype control antibody, mouse IgG2a (DAKO) were added. Secondary antibody (rabbit anti-mouse Ig horseradish peroxidase (DAKO) for the FN plate and biotin conjugated chicken anti-mouse (Life Technologies) for the FBLN-1 plate both diluted 1:500 in reagent diluent) followed by 2,2′-azino-bis(3-ethylbenzothiazoline-6-sulphonic acid) (ABTS, Life Technologies) substrate solution for the FN plate; or streptavidin-HRP followed by TMB substrate solution for the FBLN-1 plate. The FN plate was read at 405 nm while the FBLN-1 plate read at 450 nm and 570 nm on the Wallac 1420 microplate reader. The linear part of the FN standard was used to determine the concentration of FN deposited by cells. Comparison of the optical densities of the control samples to the treatment samples was used for assessing the FBLN-1 production.

### Statistical analyses

Statistical tests and graph plotting were conducted using GraphPad Prism 7 (GraphPad Software, La Jolla, California USA). A two way ANOVA with Sidak’s multiple comparisons test was used to assess significance over time, or in response to treatment and to compare immortalised and primary ASM cells. A probability (p) value of <0.05 was considered statistically significant.

## Results

### Comparison of hTERT immortalised ASM cells and primary ASM cells

**Figure 1:**
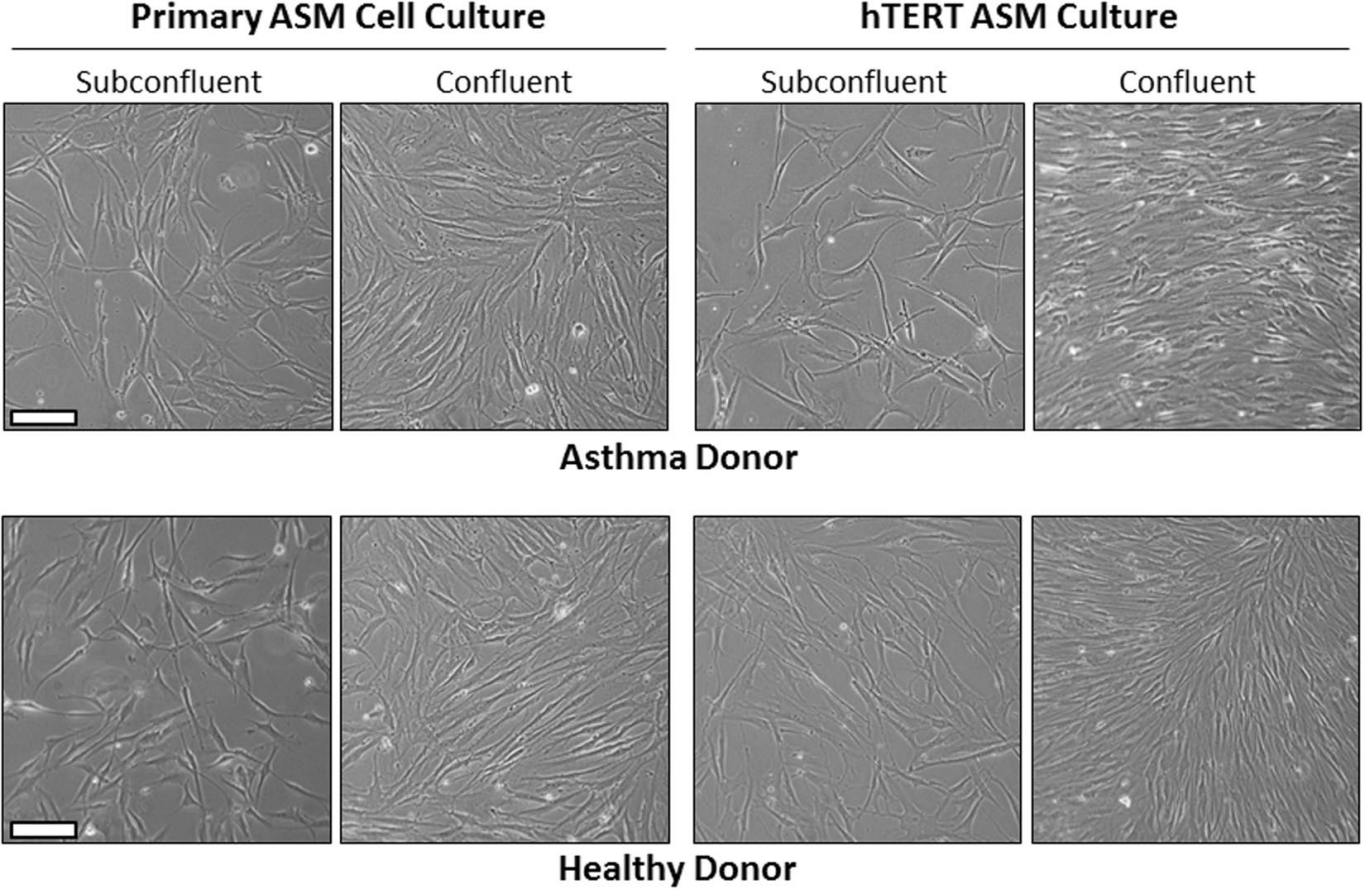
Phase contrast micrographs of primary human ASM cell cultures and corresponding hTERT immortalized human ASM cell cultures generated from an asthma donor and non-asthma donor. Typical images from subconfluent and confluent cultures grown in culture media supplemented with FBS are shown. The primary cell culture shown was at Passage 1, and the hTERT cell line shown was imaged during Passage 5 after immortalization. The white scale bar shown = 50 µm.

In our initial studies, hTERT immortalised ASM cell cultures were compared to donor-matched primary ASM cell cultures. No differences were observed in the morphological characteristics of the primary and corresponding hTERT immortalised ASM cell cultures using phase contrast microscopy (Fig. 1).

#### Proliferation

**Figure 2:**
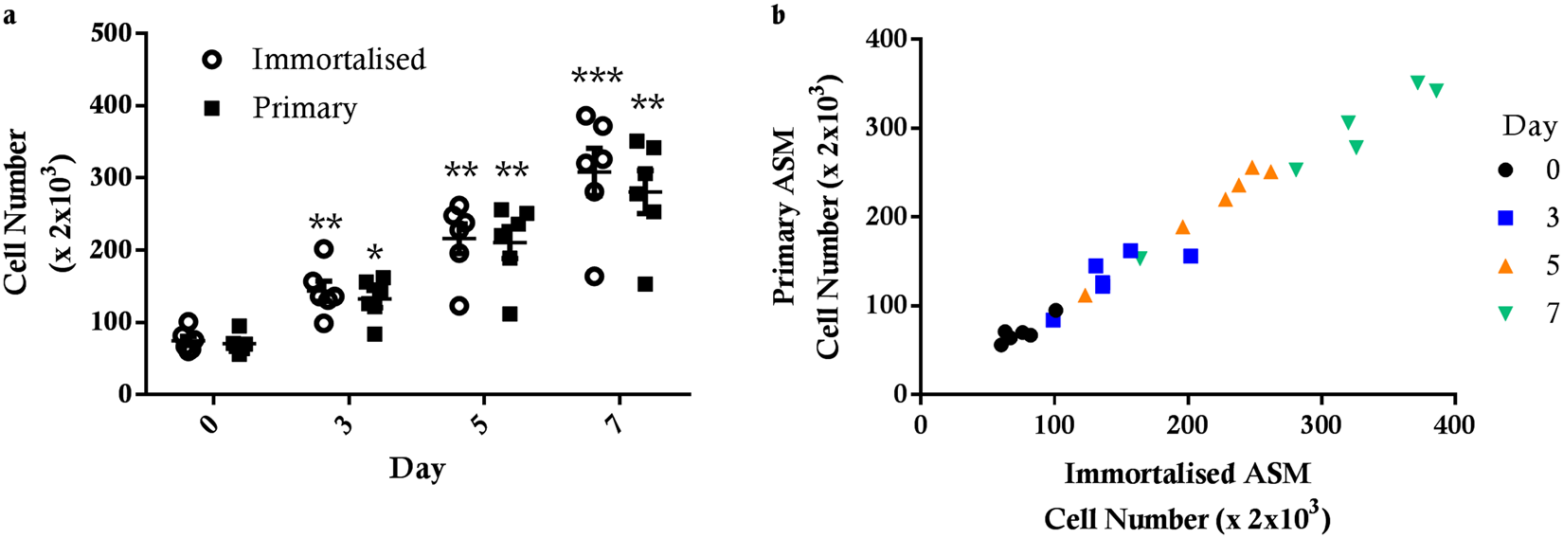
Proliferative response of hTERT immortalised and primary ASM cells. hTERT immortalised ASM (n = 6) and primary ASM (n = 6) cells from the same donor were treated with 5% FBS for 7 days. Cell number was assessed by manual cell counting. (**a**) All data are presented as the mean +/− standard error of the mean (SEM). A two way ANOVA with Sidak’s multiple comparisons test was used to assess significance over time and compare immortalised and primary cells. Significance difference compared to previous count day *p < 0.05, **p < 0.01 and ***p < 0.001. (**b**) correlation of cell counts of primary and immortalised cells from the same donor.

The number of immortalised ASM cells increased steadily over 7 days following treatment with 5% FBS (Fig. 2a). A significant increase was seen at days 3 (p < 0.01), 5 (p < 0.01) and 7 (p < 0.001), compared to day 0 and also the previous count day. Similarly, primary ASM cells responded to treatment with 5% FBS with significant increases at day 3 (p < 0.05), 5 (p < 0.01) and 7 (p < 0.01). Of note, there were no significant differences between cell numbers in hTERT immortalised and primary ASM cell cultures at any count day (Fig. 2). Moreover, the individual proliferative responses of primary and immortalised cell cultures from the same donor were directly correlated (Fig. 2b).

#### Inflammatory Profile

**Figure 3:**
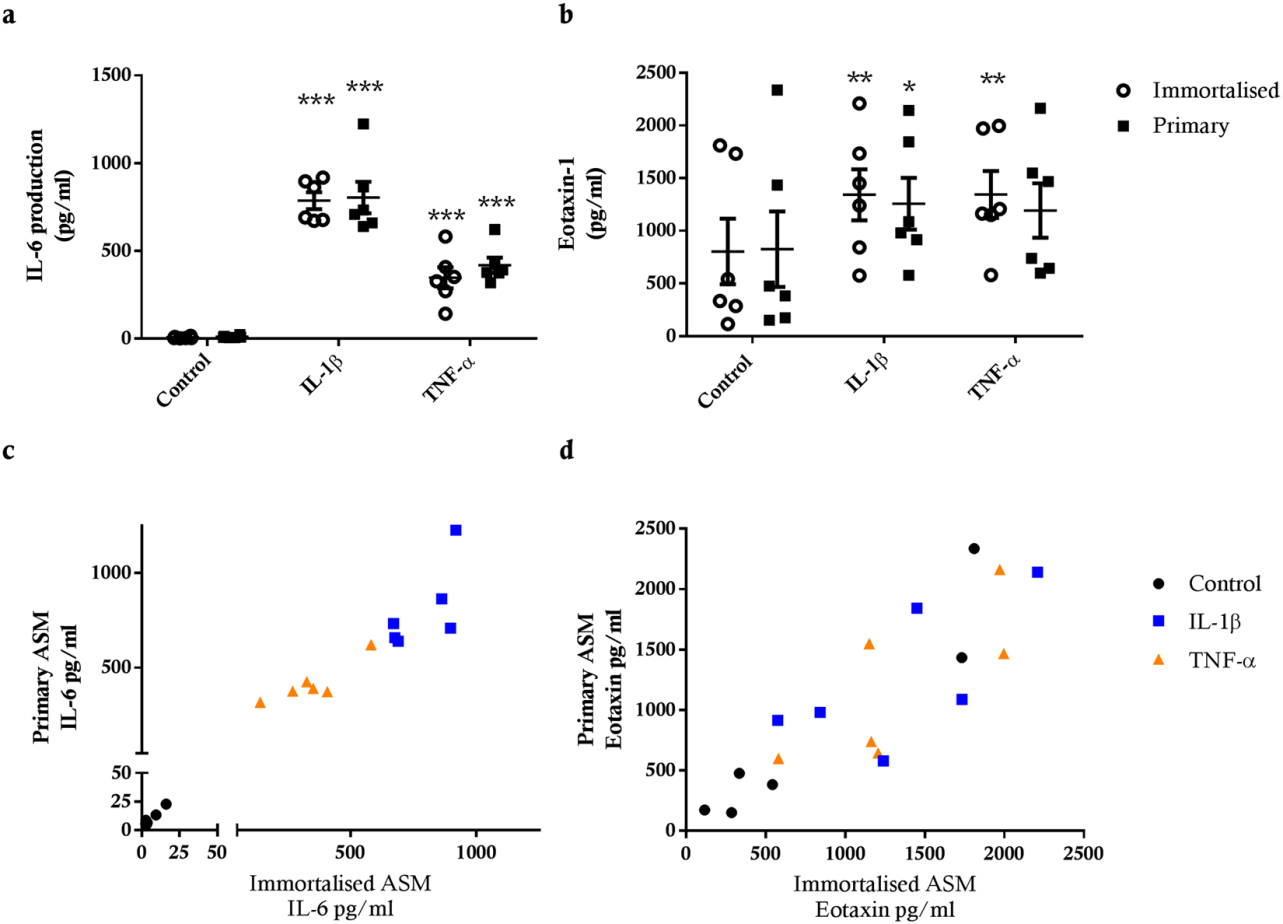
IL-6 and eotaxin-1 production from hTERT immortalised and primary ASM cells. Comparison of IL-6 (**a**) and eotaxin-1 (**b**) production from hTERT immortalised (n = 6) and primary (n = 6) ASM cells from the same donors following 24 hour stimulation with 10 ng/ml IL-1β or 10 ng/ml TNF-α. All data are presented as the mean +/− SEM. Statistical analysis was performed using a two way ANOVA with Sidak’s multiple comparisons test for changes in IL-6 or eotaxin production due to treatment and comparison of immortalised and primary cells. Significance difference due to treatment *p < 0.05, **p < 0.01 and ***p < 0.001. Correlation of IL-6 (**c**) and eotaxin (**d**) release from primary and immortalised ASM cells from the same donor.

hTERT immortalised and primary ASM cell cultures exhibited similar basal (control) levels of IL-6 and eotaxin-1 production (Fig. 3). IL-6 and eotaxin-1 release was significantly increased by IL-1β and TNF-α in hTERT ASM cell cultures (p < 0.001 and p < 0.01 respectively for both treatments) (Fig. 3a). A similar induction of IL-6 release was measured for primary ASM cell cultures (p < 0.001 for both treatments), however, only IL-1β induced a significant increase in eotaxin-1 production in primary ASM cultures (p < 0.05) (Fig. 3b). Of note, despite this difference, cytokine-induced IL-6 and eotaxin-1 release was similar in immortalised and primary ASM cell cultures. The amounts of IL-6 and eotaxin-1 produced by donor-matched hTERT immortalised and primary ASM cell cultures were not different (Fig. 3c,d).

#### Growth Factors

**Figure 4:**
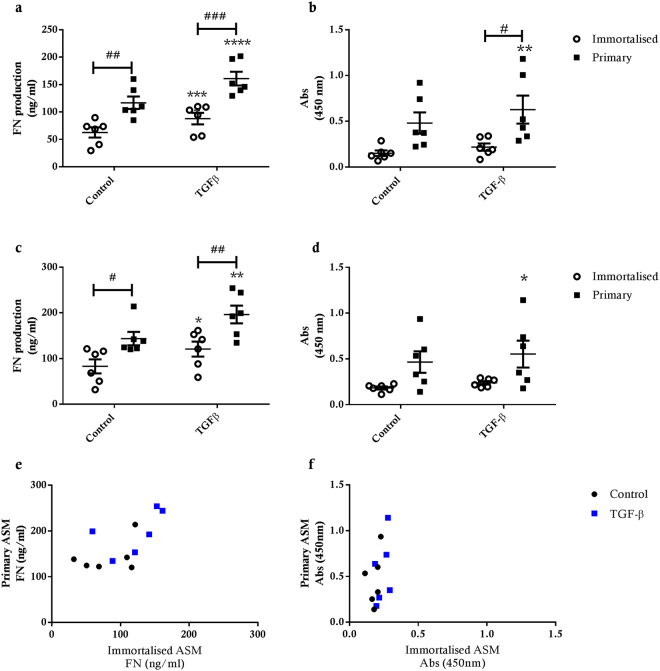
CTGF production from hTERT immortalised and primary ASM cells. Production of CTGF from hTERT immortalised (n = 6) and primary (n = 6) ASM cells following 24 hour treatment with 10 ng/ml TGF-β. (**a**) Data are presented as the mean +/− SEM of absorbance values (450 nm) in presence of CTGF Ab. A two way ANOVA with Sidak’s multiple comparisons test was used to detect differences between control and TGF-β treated groups and to compare immortalised ASM to primary ASM. Significant difference due to treatment *p < 0.05. (**b**) Correlation of CTGF production from primary and immortalised ASM cells from the same donor.

Immortalised ASM cell cultures were unique in that they showed a significant increase in CTGF production following TGF-β treatment (p < 0.05), whereas this response was not evident in the primary ASM cell cultures (Fig. 4a). Immortalised and primary ASM cell cultures derived from the same donor showed no variation in the amount of CTGF produced (Fig. 4b).

#### ECM Protein Production

**Figure 5:**
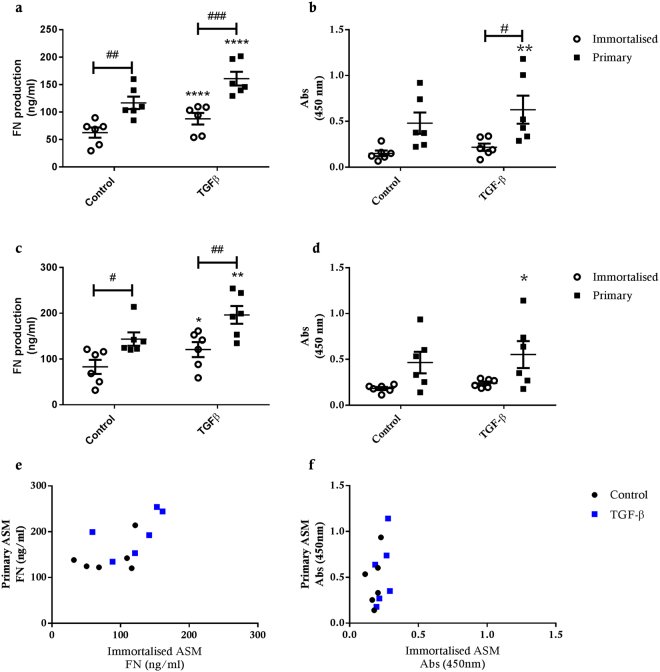
Fibronectin and fibulin-1 deposition by hTERT immortalised and primary ASM cells. FN and fibulin-1 deposition from hTERT immortalised (n = 6) and primary (n = 6) ASM cells in response to treatment with 10 ng/ml TGF-β. (**a**) FN and (**b**) fibulin-1 after 48 hours treatment and (**c**) FN and (**d**) fibulin-1 after 72 hours treatment with TGF-β. All data are presented as the mean +/− SEM. Statistical analysis performed using a two way ANOVA with Sidak’s multiple comparisons test for comparison of untreated to treated cells, to detect differences across time and also to detect significant differences between immortalised and primary cells. Significance between untreated and treated cells *p < 0.05 and **p < 0.01. Significance between immortalised and primary cells ^#^p < 0.05, ^##^p < 0.01 and ^###^p < 0.001. Correlation of FN (**e**) and fibulin-1 (**f**) deposition after 72 hours treatment with TGFβ from primary and immortalised cells from the same donor.

Both immortalised and primary ASM cell cultures exhibited significantly increased FN production following exposure to TGF-β1 (p < 0.0002, p < 0.05) (Fig. 5a and c). In contrast, we only observed a significant increase in TGF-β1-induced FBLN-1 deposition in primary ASM cell cultures (p < 0.01 for 48 h, p < 0.05 for 72 h), suggesting that FBLN-1 deposition in immortalised ASM cell cultures is refractory to TGF-β1 exposure (p < 0.05) (Fig. 5b and d). Compared to the primary ASM cell cultures, the immortalised cells produced significantly less FN in untreated (p < 0.01 for 48 h, p < 0.05 for 72 h) and TGF-β1 treated (p < 0.001 for 48 h, p < 0.01 for 72 h) conditions (Fig. 5a and c). However, there were no significant differences detected in FBLN-1 deposition between immortalised and primary ASM cell cultures under control conditions or after TGF-β1 exposure (Fig. 5b and d). Comparing donor-matched hTERT immortalised and primary ASM cell cultures directly, we observed that FN and FBLN-1 production was lower in immortalised cell lines in control conditions and after TGF-β1 exposure (Fig. 5e and f).

### Comparison of immortalised asthmatic and non-asthmatic ASM cells

Based on our observations comparing donor-matched primary and hTERT ASM cell cultures, we next determined whether previously described functional differences between primary ASM cell cultures from asthmatic and non-asthmatic donors are maintained in hTERT immortalised ASM cell cultures generated from these parent cell lines.

#### Proliferation

**Figure 6:**
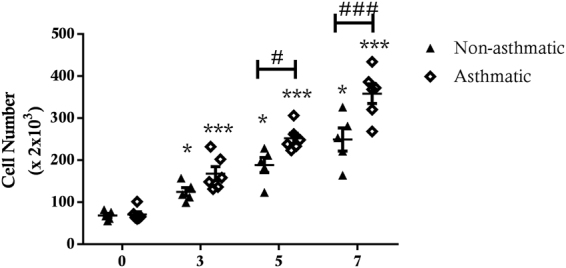
Comparison of proliferation between immortalised ASM of asthmatic and non-asthmatic patients. hTERT immortalised nonasthmatic ASM (n = 5) and asthmatic ASM (n = 6) cells were treated with 5% FBS for 7 days. Cell number was assessed by manual cell counting. All data are presented as the mean +/− SEM with statistical analysis via a two way ANOVA with Sidak’s multiple comparisons test. Analysis was performed to detect differences between each count day and asthmatic and non-asthmatic ASM cell numbers. Significant cell number increase *p < 0.05 and ***p < 0.001. Significance between immortalised asthmatic and non-asthmatic cells ^#^p < 0.05 and ^###^p < 0.001.

Immortalised ASM cell cultures from both asthmatic and non-asthmatic patients showed a significant increase in cell number over 7 days in media supplemented with 5% FBS. There was a significant increase in cell number between each count day for asthmatic (p < 0.001 for all days) and non-asthmatic (p < 0.05 for all days) hTERT ASM cells. Importantly, by day 5 (p < 0.05) and day 7 (p < 0.001) there were significantly higher numbers of cells in cultures of asthmatic hTERT ASM cell lines compared to cultures of hTERT ASM cells from non-asthmatic donors (Fig. 6). This finding indicates that a hyper-proliferative phenotype is retained in asthmatic ASM cells after hTERT immortalization.

#### Inflammatory Profile

**Figure 7:**
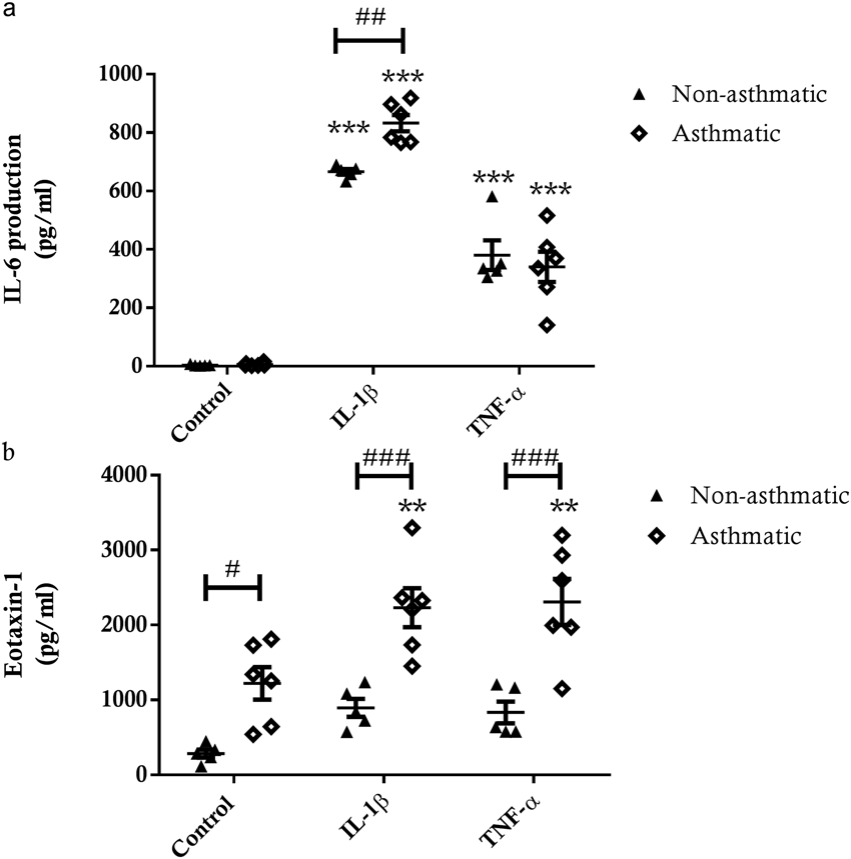
IL-6 and eotaxin-1 release from immortalised non-asthmatic and asthmatic ASM cells. (**a**) IL-6 and (**b**) eotaxin-1 release from ASM from immortalised non-asthmatic (n = 5) and asthmatic (n = 6) donors in response to 24 hour treatment with 10 ng/ml IL-1β or 10 ng/ml TNF-α. All data are presented as the mean +/− SEM. A two way ANOVA with Sidak’s multiple comparisons test was used to detect differences between asthmatic and non-asthmatic cells and treatment groups. Significance between asthmatic and non-asthmatic cells ^#^p < 0.05, ^##^p < 0.01, ^###^p < 0.001. Significance difference induced by treatment compared to control **p < 0.01, ***p < 0.001.

Although IL-6 release under basal conditions was not different in hTERT ASM cultures from asthmatic and non-asthmatic donors, under basal conditions eotaxin-1 release was significantly higher in hTERT immortalised asthmatic ASM cell cultures (p < 0.05) (Fig. 7). Compared to untreated control conditions, treatment with IL-1β and TNF-α induced a significant release of both IL-6 (p < 0.001) and eotaxin-1 (p < 0.01) from cultures of immortalised asthmatic ASM cell cultures (Fig. 7). In immortalised non-asthmatic ASM cell cultures, while IL-6 release was significantly increased in response to IL-1β and TNF-α treatment (p < 0.001), surprisingly, the increase in eotaxin-1 was not significant (Fig. 7b). Importantly, IL-1β-induced release of IL-6 was significantly greater for immortalised asthmatic ASM cell cultures compared to non-asthmatic ASM cell cultures (p < 0.01) (Fig. 7a), whereas we observed no difference in TNF-α-induced release of IL-6 in asthmatic and non-asthmatic ASM cell cultures. Furthermore, both IL-1β- and TNF-α-induced release of eotaxin-1 was markedly greater in immortalised asthmatic ASM cell cultures compared to non-asthmatic ASM cells (p < 0.001 for both treatments) (Fig. 7b).

#### Growth Factors

**Figure 8:**
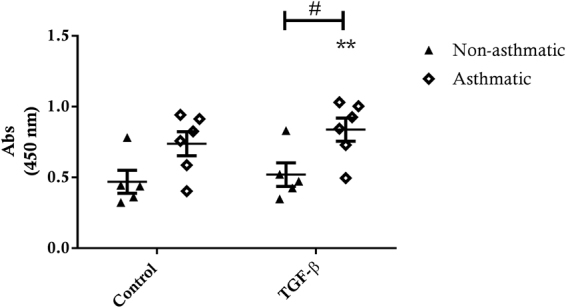
CTGF production from immortalised asthmatic and non-asthmatic ASM cells. CTGF production from immortalised non-asthmatic (n = 5) and asthmatic (n = 6) ASM cells following 24 hour treatment with 10 ng/ml TGF-β. All data are presented as the mean +/− SEM with statistical analysis via a two way ANOVA with Sidak’s multiple comparisons test. Analysis was performed between control and treatment groups and immortalised asthmatic and non-asthmatic ASM cells. Significance between control and treatment **p < 0.01. Significance between immortalised asthmatic and non-asthmatic samples ^#^p < 0.05.

There was a significant increase in CTGF production in response to TGF-β1 for immortalised asthmatic ASM cell cultures (p < 0.01) (Fig. 8). However, the same increase was not evident in the immortalised non-asthmatic ASM cell cultures. This difference manifested as greater cumulative TGF-β1-induced synthesis of CTGF by hTERT immortalised asthmatic ASM cell cultures compared to non-asthmatic ASM cell cultures (p < 0.05).

#### ECM Protein Production

**Figure 9:**
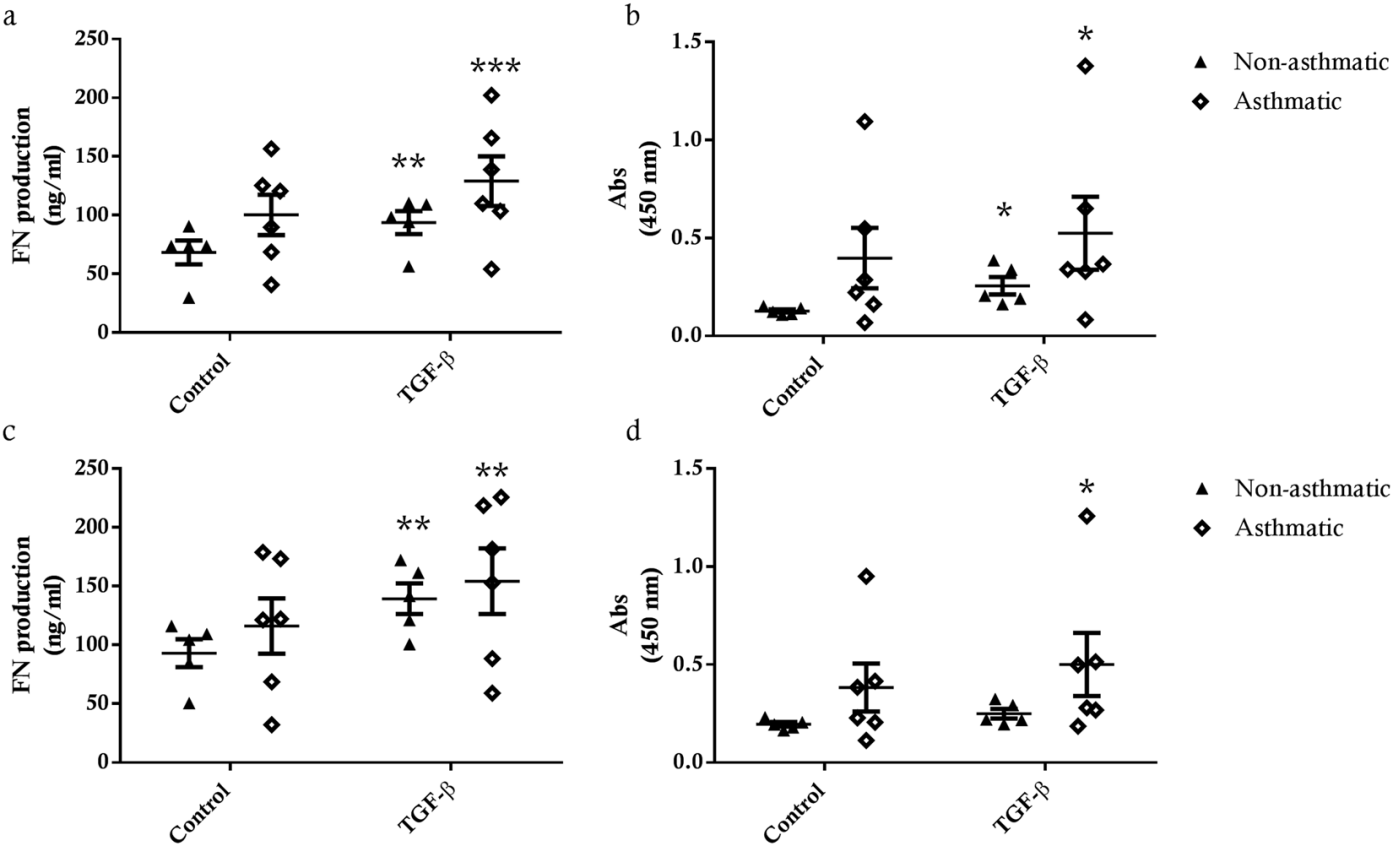
Fibronectin and fibulin-1 deposition by immortalised asthmatic and non-asthmatic ASM cells. FN and FBLN-1 deposition from immortalised non-asthmatic (n = 5) and asthmatic (n = 6) ASM cells after 48 hrs (**a** and **b** respectively) or 72 hrs (**c** and **d** respectively) in response to treatment with 10 ng/ml TGF-β. All data are presented as the mean +/− SEM. Statistical analysis via a two way ANOVA with Sidak’s multiple comparisons test was performed to test differences between control and treatment groups and immortalised asthmatic and non-asthmatic ASM cells. Significance as a result of treatment is represented by **p < 0.01 and ***p < 0.001.

TGF-β1 treatment induced an increase in FN and FBLN-1 deposition in immortalised asthmatic ASM cell cultures (FN 48 hrs p < 0.001, 72 hrs p < 0.01; FBLN-1 48 & 72 hrs p < 0.05) (Fig. 9). In immortalised non-asthmatic ASM cells, TGF-β1 also induced significant FN deposition at both 48 and 72 hours (48 hrs p < 0.05, 72 hrs p < 0.01), however, compared to control conditions, TGF-β1 only induced significant FBLN-1 accumulation 48 hrs after exposure (p < 0.05). Interestingly, for all cultures maximum FN and FBLN-1 accumulation was attained within 48 hrs TGF-β1 treatment, as there was no additional deposition at 72 hrs treatment. In contrast to enhanced release of IL-6, eotaxin-1 and CTGF, we observed no difference in the deposition of FN or FBLN-1 by immortalised ASM from asthmatic or non-asthmatic donors (Fig. 9).

## Discussion

This study shows for the first time that hTERT immortalised ASM cells provide a useful tool for characterising mechanisms relating to differences in tissue inflammatory responses in asthma, as they mirror unique hyper-secretory characteristics of primary ASM cells from asthmatic donors^13–17^. Using donor-matched parent and immortalised cell lines, we reveal that hTERT immortalised ASM cell cultures retain a similar proliferative response, inflammatory profile and growth factor production as parent primary ASM cell cultures. Despite continuity in these important pathophysiological functions, we also observed that hTERT immortalisation did not retain a full repertoire of ECM protein deposition, as immortalised ASM cells exhibited hypo- or refractory-responsiveness to TGF-β1-induced FN and FBLN1 deposition, indicating that there are some limitations in using these cells to assess all aspects of airway matrix remodelling. This study also compared immortalised ASM cells derived from asthmatic and non-asthmatic subjects, and overall, our new data demonstrate that many previously established differences in functional characteristics of ASM from asthmatic subjects are maintained after hTERT immortalisation. While we did not compare the responses of the primary asthmatic and non-asthmatic cells in this study, some of these cells had been used as part of previous studies in which differences in the responses measured in this study were reported. The current data reflect prior studies that describe hyper-proliferative capacity of primary ASM cells derived from asthmatic subjects^14,15^, as well as hyper-secretory inflammatory mediator and growth factor release of primary human asthmatic ASM cell cultures^18–22,27^. Conversely, we did not observe that hTERT immortalised asthmatic ASM cell cultures retained characteristic differences in FN and FBLN1 synthesis seen in primary ASM cell cultures from asthmatic donors^14,17,26^. Collectively, our observations demonstrate that hTERT immortalisation of human ASM cells is an effective approach to generate a sustainable and reliable *in vitro* system to dissect key elements of ASM pathobiology in asthma. In a separate study we have undertaken a systematic assessment of the method of immortalising primary ASM cells, which includes only a general assessment of functional and phenotypic properties of human hTERT ASM cell lines, but does not directly test whether hTERT immortalisation maintains the unique, heterogeneous nature inherent to ASM primary cell cultures from different donors (submitted July 2017).

Immortalised ASM cells mirrored primary ASM cell cultures in their response to a pro-proliferative stimulus, 5% FBS, inducing similar proliferation in immortalised and primary ASM over the course of a typical single cell culture passage (7 days). Immortalised cell cultures also behaved the same as corresponding primary ASM cell cultures in terms of cytokine and chemokine release with no significant variation in IL-6 and eotaxin-1 release between the two cell models. Of mention, the level of eotaxin-1 released by the primary cells in response to TNFα and IL-1β did not differ. So while the induction of eotaxin-1 was significantly different from the control levels in the immortalized ASM cells but not in the primary cells in the current data series, we do not believe this represents a differential response between these cells. It is important to note that “hTERT immortalisation” reflects the induction of a senescence resistant state, but is not associated with cell transformation per se, thus cells retain a requirement for adherence-dependent growth and are refractory to acquisition of a senescence-associated hyper-secretory phenotype^37,38^.

The production of CTGF from immortalised ASM cells was also similar to that of corresponding primary ASM cells, however, we found that the responsiveness of primary ASM cells TGF-β1-induced CTGF production was generally lower than that we have reported in previous studies^24,25^. This may be related to us employing an ELISA method for cell surface, not intracellular, detection in the current study, compared to immunoblotting assays used in earlier work. Nonetheless, basal and TGF-β1-stimulated CTGF production was similar between immortalised and primary ASM cells.

The major discrepancy we observed between immortalised and primary human ASM cell cultures was in the production of ECM proteins. FN production was significantly lower in immortalised ASM cells compared to the corresponding primary ASM cells. Similarly, there was a significant variation between immortalised and primary ASM cell cultures for FBLN-1 deposition following TGF-β1 exposure. One role of the ECM is to provide the cells with survival signals, through integrin surface receptors, to prevent programmed cell death induced following detachment from ECM (anoikis). Disruption of communication between the ASM cells and ECM elements such as FN or FBLN1 can result in cell death and alterations in the ECM can also change the growth patterns of ASM cells^9,13,39^. It is possible that the process of hTERT immortalisation of ASM cells may disrupt anoikis programming, thus homeostatic requirements for ECM deposition may be affected. Indeed endothelial cells that acquire anoikis resistance display decreased expression of fibronectin and other ECM proteins and an alteration in their integrin repertoire^40^. Of note integrin β1 is a downstream target of hTERT^41^ suggesting disruption of this integrin may occur in the immortalised ASM cells.

The ECM also has a role in mediating proliferation and the production of cytokines, chemokines and growth factors^13,42–44^. Since these characteristics were not different between immortalised and primary ASM cells, the altered deposition of ECM proteins in immortalised cells does not appear, in this case, to be a critical determinant of proliferation or inflammatory mediator release. Given that FN was induced by TGF-β1, albeit at a lower level than seen in the primary cells, it is possible that the reduction in ECM expression in the immortalised ASM cells did not reach a threshold necessary to influence these parameters. Alternately, other pathways, such as mammalian target of rapamycin (mTOR)/Akt pathway, peroxisome proliferator-activated receptor gamma (PPAR γ) or mitogen-activated protein kinases (MAPKs)^45–51^, may maintain proliferative function and inflammatory profiles in immortalised ASM cells.

Once the validity of the immortalised ASM cells as a model for primary ASM cells had been confirmed we also undertook a comparison of hTERT immortalised ASM cell cultures from asthmatic and non-asthmatic donors. Our results showing enhanced proliferation of immortalised asthmatic ASM cells are consistent with prior studies that compared primary asthmatic and non-asthmatic human ASM cell cultures^6,7^. Immortalised asthmatic ASM cells only had significantly increased IL-1β-induced IL-6 production compared to non-asthmatic cells, but there was no difference in TNF-α induced IL-6 production. Several studies report that TNF-α exposure increases production of IL-6 by human ASM cells^19,21^, but, there are few studies that compare the IL-6 production between asthmatic and non-asthmatic ASM cells, with only one study reporting that IL-6 release was greater from asthmatic compared to non-asthmatic ASM cells after rhinovirus exposure^22^. There were greater amounts of basal and IL-1β- or TNF-α-induced eotaxin-1 produced by immortalised asthmatic ASM cell cultures compared to immortalised non-asthmatic ASM cells. This finding is fully consistent with previous studies that report eotaxin-1 hypersecretion in primary asthmatic ASM cell cultures^23^. Our observation using immortalised cultures that CTGF production is greater in TGF-β1 treated asthmatic ASM cells compared to non-asthmatic ASM cells is also consistent with our previous studies^24,25^ in which we illustrated the importance of the asthmatic ASM in regulating the enhanced angiogenesis in the airways, through release of CTGF and other factors^12,24,33,52,53^.

In contrast to the release of cytokines, chemokines and growth factors, our comparison of ECM protein deposition between immortalised asthmatic and non-asthmatic ASM cell cultures revealed no significant differences. These findings are not reflective of previous work, using primary ASM cells, in which TGF-β induced FN biosynthesis is greater in ASM from asthmatic donors compared to those from non-asthmatic donors^26^. As already noted, this apparent disparity between the data in the current study and previous reports suggests that capacity for FN and FBLN-1 deposition is affected by the hTERT immortalisation process. As a caveat, we cannot specifically rule out the possibility that for the group of individual primary cell lines used to generate hTERT immortalised ASM cell lines in this study, there were also no differences FN and FBLN-1 output, as we did not measure this in the current study, and some degree of variability can exist. However, our previous work with some of these cells suggests that the latter possibility is unlikely.

A limitation of this study is that we did not directly monitor hTERT incorporation in the ASM cell lines tested. Moreover, we did not determine if there are any differences in the expression of ectopic hTERT in immortalised ASM cells from asthmatic and non-asthmatic patients. Of note, the immortalisation process uses lentiviral vectors to transduce primary ASM cells and promote homologous recombination of copies of the hTERT. To reduce inter-culture and inter-cell disparity in hTERT incorporation, cells are subjected to severe antibiotic selection, thus only those cells that incorporate multiple copies of hTERT will exhibit sufficient resistance to selection with high concentrations of G418 antibiotic. Nonetheless, we cannot rule out the possibility that there may be differences between hTERT cell lines or individual cells in hTERT cell lines due to disparate ectopic hTERT expression. The cell lines tested were also not expanded from single clones but rather represented a mixed population of cells. Our studies did not address potential effects that differing expression of hTERT may specifically have on proliferation rates, inflammatory and growth factor production, and ECM deposition.

In conclusion, this study validates hTERT immortalised ASM cell lines as a reliable and sustainable resource for use in future investigation to elucidate the role of the ASM in asthma pathophysiology. hTERT immortalised ASM cells retain proliferation, inflammatory cytokine and chemokine release and growth factor production characteristics of the primary ASM cell cultures from which they are generated. However, some caution is needed with respect to capacity for biosynthesis of FN and FBLN-1; though expression of these ECM proteins is retained in immortalised ASM cell lines, the capacity for biosynthesis san responsiveness to pro-fibrotic growth factor exposure appears to be dampened by hTERT immortalisation. In the future, the use of increasingly sensitive methods for the detection of ECM proteins may provide insight about appropriate use of hTERT immortalised ASM cells in this area of research.

Overall, this study has provided the foundation for the introduction of new valuable immortalised ASM cell lines from asthmatic and non-asthmatic human donors. This initial validation will pave the way forward enabling future studies using more complex *in vitro* systems that could, for example, involve 3D bio-printing, with co-culture of immortalised ASM with fibroblasts and epithelial cells, to generate a structure that more closely reflects the *in vivo* airway for the investigation of mechanisms controlling asthma pathophysiology.
